# Towards cash transfer interventions for tuberculosis prevention, care and control: key operational challenges and research priorities

**DOI:** 10.1186/s12879-016-1529-8

**Published:** 2016-06-21

**Authors:** Delia Boccia, Debora Pedrazzoli, Tom Wingfield, Ernesto Jaramillo, Knut Lönnroth, James Lewis, James Hargreaves, Carlton A. Evans

**Affiliations:** London School of Hygiene and Tropical Medicine, Faculty of Epidemiology and Population Health, Keppel Street, WC1E 7HT London, UK; Infectious Disease & Immunity and Wellcome Trust Centre for Global Health Research, Imperial College, London, UK; The Monsall Infectious Diseases Unit, North Manchester General Hospital, Manchester, UK; World Health Organization, Global Tuberculosis Programme, Geneva, Switzerland; Department of Public Health Sciences, Karolinska Institutet, Stockholm, Sweden; London School of Hygiene and Tropical Medicine, Faculty of Public Health and Policy, London, UK; Innovation For Health And Development (IFHAD), Universidad Peruana Cayetano Heredia, Lima, Peru

**Keywords:** Tuberculosis, Social protection, Cash transfer, Implementation, Conditionality, Targeting, Co-financing

## Abstract

**Background:**

Cash transfer interventions are forms of social protection based on the provision of cash to vulnerable households with the aim of reduce risk, vulnerability, chronic poverty and improve human capital. Such interventions are already an integral part of the response to HIV/AIDS in some settings and have recently been identified as a core element of World Health Organization’s End TB Strategy. However, limited impact evaluations and operational evidence are currently available to inform this policy transition.

**Discussion:**

This paper aims to assist national tuberculosis (TB) programs with this new policy direction by providing them with an overview of concepts and definitions used in the social protection sector and by reviewing some of the most critical operational aspects associated with the implementation of cash transfer interventions. These include: 1) the various implementation models that can be used depending on the context and the public health goal of the intervention; 2) the main challenges associated with the use of conditionalities and how they influence the impact of cash transfer interventions on health-related outcomes; 3) the implication of targeting diseases-affected households and or individuals versus the general population; and 4) the financial sustainability of including health-related objectives within existing cash transfer programmes. We aimed to appraise these issues in the light of TB epidemiology, care and prevention. For our appraisal we draw extensively from the literature on cash transfers and build upon the lessons learnt so far from other health outcomes and mainly HIV/AIDS.

**Conclusions:**

The implementation of cash transfer interventions in the context of TB is still hampered by important knowledge gaps. Initial directions can be certainly derived from the literature on cash transfers schemes and other public health challenges such as HIV/AIDS. However, the development of a solid research agenda to address persisting unknowns on the impact of cash transfers on TB epidemiology and control is vital to inform and support the adoption of the post-2015 End TB strategy.

## Background

The World Health Organization (WHO) estimates that between 2000 and 2014, 43 million lives have been saved as a result of effective tuberculosis (TB) diagnosis and treatment [1]. Despite this, the decline in TB incidence remains slow (1⋅5 % decrease in global estimated TB incidence rates annually) contributing to the persistent high global burden [1]. This slow decline suggests that a biomedical approach will be not sufficient to end the TB epidemic and achieve TB elimination in the near future. There is increasing consensus that interventions tackling the social determinants of TB may play an important role in the fight against this disease [2, 3]. This vision is reflected in WHO’s new, post-2015, End TB Strategy which identifies universal health coverage, social protection and actions on social determinants as key elements of the global response to TB. The strategy includes also a target that no TB affected household should experience catastrophic costs due to TB [4].

**Fig. 1:**
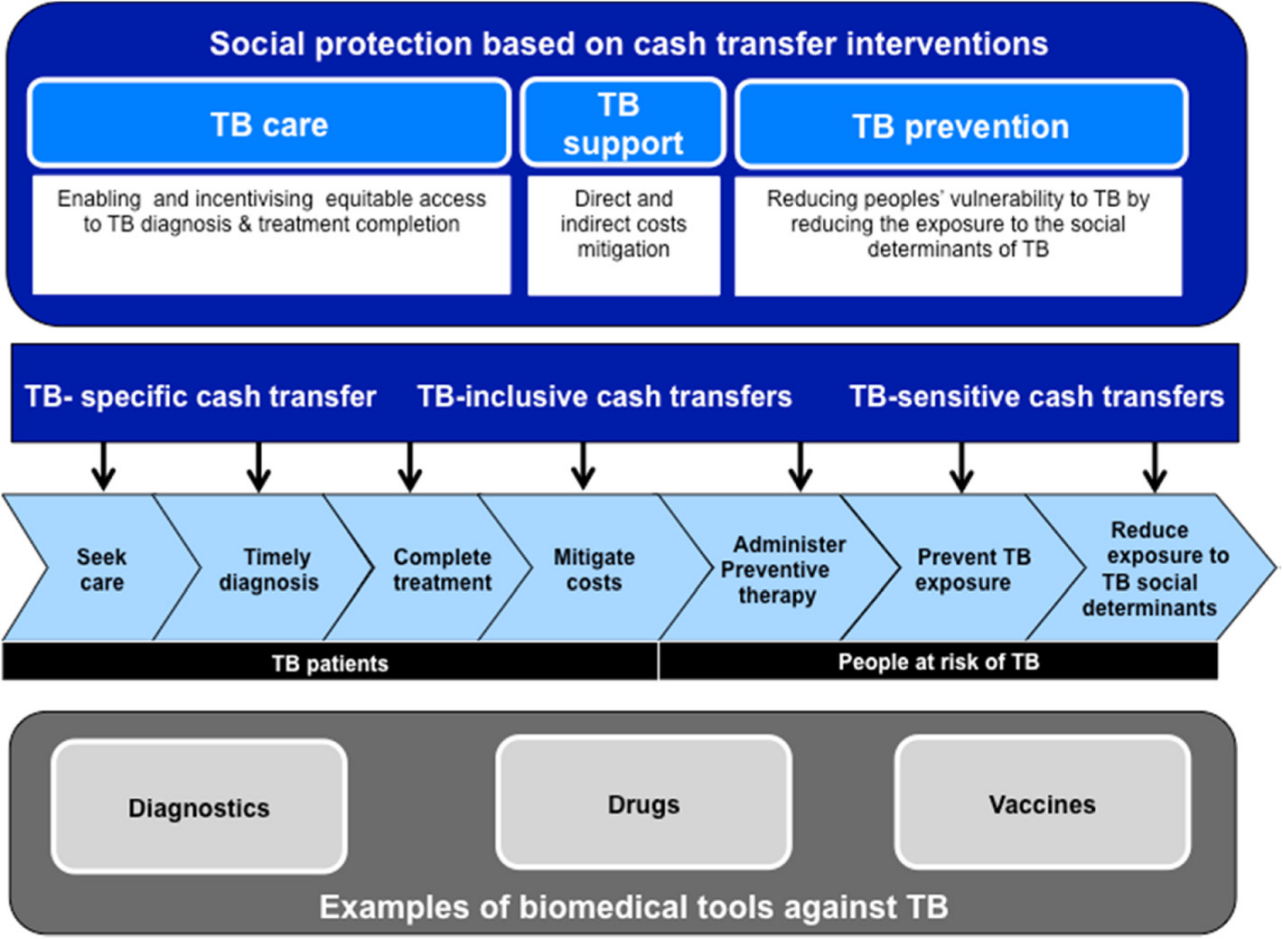
The role of cash transfer interventions in TB care, support and prevention: a conceptual framework. Cash transfer interventions can potentially enable equitable access to TB services (TB care), mitigate TB related-costs (TB support) and reduce TB susceptibility among people at risk (TB prevention) through impact on living conditions and the expected impact on important risk factors for TB (such as under-nutrition, HIV, inadequate housing conditions, etc.). Cash transfer interventions can be TB-specific, TB-inclusive or TB-sensitive depending on the target population. The grey box shows examples of biomedical tools against TB included in this diagram to provide a comparative synthesis of the current approach to TB care and prevention at various stages of the causal pathway. Their inclusion in this framework also aims to emphasise the importance of a multi-disciplinary response to TB based on an integrated biomedical and structural approach

Social protection has been defined as a range of policies that enable people to cope with and recover from risks and adversities, with the objective of achieving poverty reduction and sustainable and inclusive economic growth [5]. A common form of social protection are cash transfer schemes that provide cash to poor population groups to reduce their vulnerability and impoverishment. Cash transfers can be given unconditionally or conditionally. In this latter case, recipients are required to take specific behavioural, education or health actions prior to cash being transferred [6, 7]. Due to their proven impact on human and financial capital [6, 8–10], cash transfers are now an integral part of the response to child malnutrition, maternal health, and HIV/AIDS in many settings [11–14] and have been hypothesised to contribute to TB elimination by enhancing TB prevention, enabling better access to TB diagnosis and treatment and mitigating the catastrophic TB-related costs incurred by TB-affected households (Fig. 1) [15, 16].

The inclusion of social protection within the End TB strategy represents a clear shift in the response to the epidemic of TB, albeit not entirely new: since early 2006 poverty reduction initiatives have been consistently embedded within the TB control strategy [17] and in the past decade many countries have included financial support for TB patients as part of their core response to TB [18]. The emphasis on social protection and Universal Health Coverage has been enhanced further in the latest End TB Strategy; however, significant unknowns remain both in terms of the impact of cash transfer interventions as well as the operational challenges associated with their implementation. We attempted to address the first knowledge gap with a review published in 2011 [15].

In this paper we aim to address the second gap by providing TB control implementers and other stakeholders with an up to date overview of some key aspects to consider when planning cash transfer interventions as part of the national TB programme response. Much of our operational appraisal is informed by three resources:

- the existing literature on cash transfers (mainly from HIV/AIDS);
- a more detailed appraisal of the evidence included in our initial review [15] to investigate the extent to which the selected cash transfer schemes could be adopted and adapted to become useful to support TB elimination. Specifically, interventions were assessed through a grid of operational questions characterising their: 1) setting; 2) feasibility, defined as practical and ethical aspects that make their implementation to enhance TB prevention, care and support realistic [19]; and 3) financial and impact sustainability (i.e. their costs and potential for such interventions to maintain their impact). These operational questions were drawn from the list of criteria for the assessment of complex public health interventions as suggested by [20, 21]
- Our extensive discussion over the years through several expert consultations with policy makers and implementers, social protection experts as well as civil society representatives [22, 23].

In this paper - after a brief overview of the known impact of cash transfers on health outcomes, including TB - we discuss the most important issues emerged through our operational appraisal, including: 1) approaches to the implementation strategy; 2) the role of conditionality; 3) the targeting options; and 4) sustainability, For each of these critical areas, we provide an overview of the most challenging aspects in the light of TB epidemiology and control and identify possible interim solutions. We conclude by drawing a roadmap for advancing the research and the policy agenda.

## Discussion

### Evidence of the health impact of cash transfers

Cash transfers are already an established element of the global response to health challenges across the world, even if their impact on health-related outcomes has been only partially demonstrated: several large reviews [9, 24, 25] concluded that conditional cash transfers have an impact on improving beneficiaries health behaviours, the magnitude of which seems to vary across countries and initiatives. The impact on health outcomes is less consistent, with evidence suggesting some effect on improving nutritional status, child growth and adult morbidity status, but not on maternal health, malaria and diarrhoea [24]. These results are consistent with what is also known for HIV/AIDS: while there seems to be a positive impact on sexual behaviours [11], the impact on actual HIV outcome has been documented only in one study so far [26].

With regards to TB, evidence is highly inconsistent: a recent Cochrane review on the impact of incentives and enablers on TB treatment adherence identified only 12 eligible trials, of which 10 took place in the US (and mainly looking at special groups such as adolescents, injection drug users, homeless and prisoners), one in Timor-Leste and one in South Africa [27]. The review concluded that material incentives and enablers may have some impact on clinic attendance, but there is currently insufficient evidence to conclude if they can improve long term adherence to TB treatment [27]. In contrast, evidence from Peru [28], Brazil [29], Ecuador [30], and Moldavia [31], showed significantly higher cure rates among TB patients receiving financial enablers compared to those who did not. These studies examined the impact of cash transfers for people ill with TB. Other studies have demonstrated, using an ecological cross-national analytical approach, that higher national general social protection spending is associated with lower national TB rates [32, 33].

While encouraging, a more thorough analysis of the evidence above suggests that the use of financial incentives to enhance TB prevention, care and support remains fragmented, rarely formally embedded into broader social protection schemes, and supported by a weak and inconsistent body of evidence with large variability in the design, implementation and evaluation strategies adopted. The weakness of impact evidence is further exacerbated by the persisting knowledge gaps on the operational challenges surrounding the implementation of cash interventions in the context of TB prevention, care and control. Important impact and operational lessons (mainly in terms of impact on health seeking behaviours and need for conditionality) could be drawn from the HIV/AIDS experience, nonetheless significant unknowns concerning conditional cash transfers remain even for HIV/AIDS including: programme design and monitoring, human rights concerns, potential for perverse incentives; availability of supply-side complements; and scale up, sustainability and costs [12, 13, 34, 35]. Furthermore, the use of cash transfers to enhance TB prevention, care and support may present some specific challenges that may be worth exploring with a separate operational research agenda.

### Overview of key operational challenges and potential solutions

#### Implementation strategy

##### Challenges

Establishing that cash transfers can effectively impact TB control is only the first step in deciding whether to, and how to, implement a cash transfer programme. Consistently with what suggested for HIV/AIDS [13, 35], three implementation models can be conceptualised in the context of TB elimination: TB-specific, TB-sensitive and TB-inclusive. TB-specific interventions are interventions targeted at TB-affected households/individuals with the precise intent of improving a number of TB indicators. TB-sensitive interventions are interventions that can potentially affect TB epidemiology and control by targeting people at high risk of TB. Finally TB –inclusive are interventions in which having TB or being a member of an household affected by TB is one of the inclusion criteria for the programme, albeit not the only one (Table 1). Each of these models is characterised by a different scope, targeting strategy, extent of integration with the local social protection platforms, and advantages and disadvantages that are likely to be highly setting-specific (Table 1).

**Table 1:** Implementation strategies: working definitions for TB

	Definition	Examples	Advantages	Disadvantages
TB-specific initiatives	Cash transfer interventions explicitly targeting TB-affected individuals and/or households with the intent of addressing a specific TB care and prevention issue [35].	ISIAT [28] and CRESIPT Project in Peru [46] Voucher intervention in South Africa [43, 44]	They may represent the only option in contexts where existing social protection schemes have limited resources hampering the further expansion of their scope (i.e. the inclusion of TB control objectives) They may be more suitable in contexts where specific vulnerable groups are involved and/or treatment adherence support or costs mitigations interventions are to be prioritised	TB control programs staff may not have the competence and resources to manage these extra activities They may be perceived as stigmatising
TB-inclusive initiatives	Cash transfer schemes that are not limited to TB-related issues but include TB disease amongst their eligibility criteria.	Temporary Disability Grant in South Africa addressing people temporarily unable to work, including people living with TB disease and MDR-TB cases in particular	Same as the TB-specific initiatives They may represent a good compromise between TB-sensitive and TB specific to minimise the respective disadvantages	The may be still perceived as stigmatising. Further the impact of the intervention may be diluted across other health outcomes
TB-sensitive initiatives	Cash transfers interventions not specific to TB patients but that could have an impact for TB patients or for TB prevention because they target groups and/or people at high risk of TB and vulnerable to deeper impoverishment due to its consequences [35].	Bolsa Familia conditional cash transfer scheme in Brazil that may occasionally enrol TB patients not because of their health status, but because they meet the enrolment poverty profile applied by the programme [41]	They may represent the most efficient way to optimise existing resources They may be the best choice in contexts where TB incidence is not going down despite the good performance of the local TB control programmes in terms of percentage of case finding and treatment success rates They reduce the risk of stigmatisation of TB-patients	Making them more inclusive for people at risk of TB may interfere with their performance and affect their budget, especially in countries where these schemes are already run with limited resources Government-run schemes may be reluctant to address public health problems as their main objective remain fundamentally to address poverty Government-run schemes may be reluctant to address TB over other public health priorities

It is likely that - as for HIV/AIDS – the most cost-effective approach will be to adapt existing cash transfer schemes to make them more TB-sensitive, rather than creating or scaling up de novo programmes. However, it remains unclear as yet how existing national TB programmes will build effective and cost-effective partnership models with the institutions in charge of the design, implementation and evaluation of governmental cash transfer programmes.

Furthermore, the potential to implement TB-sensitive cash transfer schemes seems to follow an “inverse care law” pattern [36] by which most impoverished countries highly affected by TB are least able to afford cash transfer implementation or expansion to encompass TB objectives without jeopardizing existing TB health care provision coverage or quality. For example, pilot cash transfer schemes launched in Zambia between 2003 and 2004 [37–39], were effective in helping destitute HIV-affected households withstand the economic impacts of AIDS. However, these schemes have not been absorbed into the country’s welfare system [38], may have limited capacity for scale-up or sustainability [37] and may lack the human and financial resources for monitoring and evaluation. This limits the potential of these schemes to become TB-sensitive or TB-inclusive by including TB care, prevention and/or support objectives.

Even in countries where comprehensive government-led social protection programmes are in place, there may be obstacles to make these programmes more inclusive for TB patients. For example, South Africa has extensive cash transfer schemes, including the Temporary Disability Grant in which TB patients are eligible to receive cash when unable to work. While the scheme was initially widely implemented, over the years the number of TB patients eligible for the scheme has been reduced to include only patients with multi-drug resistant TB (MDR-TB) and/or severe TB-related complications [40]. The reasons for this reduction included lack of evidence of the impact on TB care and prevention, insufficient allocated resources to cope with the growing number of TB-HIV co-infected patients applying for the grant, the increasing roll-out of anti-retroviral therapy, and the anecdotal reports of fraud by healthy individuals buying sputum samples from TB patients to falsely receive a diagnosis of TB and thus qualify to receive (or continue to receive) the benefits [22].

A different picture emerges from Brazil. Experts of the Bolsa Familia Programme, the Brazilian conditional cash transfer programme for impoverished households [41], have recently argued that, in Brazil, TB is serious enough but also infrequent enough to incorporate TB into the existing Bolsa Familia logistical and financial capacity, thereby becoming more TB-inclusive or TB-sensitive [Soares S. personal communication]. Furthermore, the preliminary evidence of the impact of Bolsa Familia on major public health issues, including TB, are encouraging [42]. Nonetheless the operational challenges of this synergy have not yet been fully explored.

Choosing a TB-specific format may appear to be simpler from an operational perspective but it may cause a number of implementation issues in terms of management and delivery of financial incentives from the TB care providers. Such issues emerged from the process evaluation of an intervention in South Africa [43]: the trial results suggested a small, but not statistically significant effect of the provision of vouchers on TB treatment success rates in the intervention communities [44]. However, fidelity to the intervention was low with approximately 36 % of eligible patients not receiving any voucher [44]. Such low fidelity was attributed mainly to two factors: first, nurses modified the delivery of the vouchers, mainly by arbitrarily deciding who was eligible to receive the incentive and also rejecting the trial eligibility criteria, which were perceived by the nursing staff to be inequitable. Secondly, from the nurses’ perspective, it was logistically easier to give all the vouchers in one batch at the end of the month. This practical ground-level implementation meant that some patients were obliged to come back to the clinic more than once to collect the voucher, a situation that was often unfavorable for these patients. Furthermore, the coordination of delivery of vouchers to clinics and collection of vouchers from local shops required considerable organization and a dedicated staff complement [43].

##### Solutions

In contrast to HIV/AIDS policy [35, 45], there is insufficient evidence to advocate for the adoption of a TB-sensitive approach over a TB-inclusive or TB-specific approach. However, the choice between TB-sensitive, TB-inclusive or TB-specific interventions may be guided by a preliminary programmatic assessment of both the TB epidemic profile and social protection environment based on a preliminary list of questions suggested in Table 2. This programmatic assessment should help countries to evaluate whether the TB control objectives can be realistically incorporated into existing schemes and how this can be best achieved. Ideally, National TB control programmes can have a key role in this initial assessment because they are well placed to understand how cash transfer interventions can be best tailored to TB patients and/or people at risk for TB. Ministries of Finance and Development may then advise on the foreseen issues such as the operational, financial and ethical barriers associated with the implementation of either a TB-sensitive or a TB-specific intervention.

**Table 2:** Summary of suggestions and research priorities for the operational challenges discussed

Operational challenges	Interim solutions	Research priorities
Implementation strategy	1. Preliminary programmatic assessment of the TB-epidemiology profile and existing social protection environment based on structured framework including the following: • What population group is most affected by TB? • What are the barriers that prevent people from accessing the TB care services and completing treatment? • What are the socioeconomic consequences of TB and TB care on TB affected households? • What social protection schemes are in place? What population group they target? What geographic areas they cover and how they overlap with TB distribution? What is the proportion of TB affected-individuals/households reached by these schemes based on their enrolment criteria? How these schemes could be made more inclusive for TB-patients? 2. Ideally start with interim, relatively small, TB-specific interventions, aimed to address a specific TB control indicator to generate impact evidence and operational lessons.	1. To design a programmatic assessment tool to support countries in choosing the best implementation strategy based on the TB epidemic profile and social protection features. 2. To undertake an inventory of all existing social protection initiatives somehow linked or linkable to TB control run at governmental and non-governmental level, then to identify the most promising initiatives to undergo impact and operational evaluation. 3. To create a network of impact and process evaluations from different countries so to have an overview of what works, where and why and share methodological and programmatic lessons. This could require approach, prioritising first TB-specific initiatives and natural experiments or quasi-experimental methods. 4. To develop and apply metrics to measure economic impact of TB for households 5. To develop innovative and rapid impact evaluation techniques, including mathematical modelling.
Conditional vs Unconditional	1. Undertake qualitative studies among intervention recipients to access the appropriateness of the conditions proposed and potential barriers to compliance. 2. If strict conditionality is deemed unfeasible or counterproductive, attempt the use of “soft” forms of conditionality. • Do not reduce the transfer size or decline eligibility only after several months of non-compliance with the behavioural requirements. • Do apply conditionality only for behaviour requirements that are simple to meet (i.e. attending TB education workshops).	1. Identify key TB-control related behaviours that are more likely to be affected by the use of conditionalities. 2. Explore if and how conditionality compliance is influenced by the size of cash transfer, the frequency of cash transfers, other psychosocial and behavioural determinants, TB status and stage of disease. 3. Identify strategies to effectively and cost-effectively monitoring these conditionalities.
Targeting approach	They are likely to differ depending on settings. Use multiple criteria based on poverty criteria and a risk score for TB.	1. Verify targeting accuracy through qualitative assessment and TB surveys among the cash recipients; 2. Assess the effectiveness and cost-effectiveness of different targeting strategies;
Sustainability	Identify multiple donors, domestic and international, addressing specific costs of the intervention.	1. Extended cost-effective analysis (ECEA) to evaluate costs against TB costs mitigation at household, community and country level 2. Explore co-financing mechanisms

Given the limited operational evidence on the feasibility of TB-sensitive cash transfer interventions it may be more appropriate to establish initially TB-specific cash transfer interventions run by national TB control programs and co-implemented with a governmental or non-governmental organization, with the intent of addressing certain TB-specific issues such as poor case-finding, low cure rates or TB clustering in high-risk groups (e.g. homeless, drug abusers, indigenous populations). While the long-term objective will be most likely to move towards a TB-sensitive approach, TB-specific initiatives can still be used as a valid starting point to gain insight and knowledge on the expected TB impact of these interventions as well as the costs and other important aspects to be considered when designing and implementing cash transfer interventions for TB control purposes (whether conditional or unconditional). An example of TB-specific intervention is the recent evaluated trial that in South Africa providing vouchers to TB patients [43]. Another example, the CRESIPT study in Peru, is currently under impact evaluation and summarised in Table 3 [46].

**Table 3:** An example of TB-specific cash transfer intervention: the CRESIPT study in Peru [46]

An example of a TB-specific cash transfer program was the ISIAT (Innovative Socioeconomic Interventions Against TB) project in Peru, which offered an integrated multidisciplinary community and household socio-economic intervention to TB-affected households, including food and cash transfers, microcredit, microenterprise and vocational training [28]. The results of this pilot study have informed the design of the subsequent 6-year CRESIPT (Community Randomized Evaluation of a Socio-economic Intervention to Prevent Tuberculosis) project, a community randomised study. CRESIPT aims to provide rigorous evidence of the impact of integrated social support and conditional cash transfers on: sustained cure in TB patients; prevention of TB in household contacts; and TB rates in the wider community. CRESIPT is being preceded by an on-going pilot phase to implement and refine the complex socioeconomic intervention in 32 communities, assess its impact on TB chemoprophylaxis completion, and assess its acceptance through a process evaluation.
Through engagement with participants, the national TB program and a civil society of ex-TB patients, the CRESIPT pilot developed its conditional cash transfer scheme with amounts that were perceived to be too small to affect participants’ autonomy in decision-making and large enough to reduce poverty-related TB risk factors [65]. Conditional cash transfers were provided to patient households for: i. screening for TB in household contacts and MDR-TB in patients; ii. adhering to TB treatment and chemoprophylaxis; and iii. engaging with CRESIPT social activities (household visits and participatory community meetings consisting of educational workshops and TB Clubs). A patient with non-MDR TB receiving six months of anti-TB treatment and completing all conditions optimally could receive cash transfers up to a value of US$ 230.
TB-affected households participating in the intervention received an average of US$ 183 over the course of treatment for the compliance to the conditional requirements. This amount aimed to be similar to, and thus potentially mitigate, the average TB-affected households’ direct costs of “free” TB care (i.e. TB-related costs of additional food, transport, medicines, and clinical consultations equalling approximately 10 % of an average household’s annual income). The cost of the CRESIPT pilot’s socioeconomic intervention were <10 % of overall costs of treating a TB patient with non-MDR TB in the local Peruvian setting (WHO 2014 http://www.who.int/tb/dots/planning_budgeting_tool/overview.pdf). Expert opinion suggested that an intervention that increased a National TB Programme’s budget by 50 % and led to a 33 % reduction in TB incidence would likely be adopted by governments [71, 72]. The CRESIPT pilot cash transfer intervention cost considerably less than 50 % of the per patient national TB budget, even including project staff.
An impact assessment to evaluate the effect of the CRESIPT pilot intervention on equitable access to TB treatment and prevention demonstrated improvement in treatment outcomes for patients and uptake of TB preventive therapy for the TB patients’ household members [73]. A process evaluation of the pilot suggested that: the project is likely to be sustainable due to involvement of patients and ex-patients as facilitators; there has been effective and synergistic cross-sectoral collaboration with the National TB Programme; and there is a perception from participants that the conditional cash transfers were patient-centred and empowering, especially for women. On the other hand the preliminary results of the process evaluation have shown challenges including: hidden bank charges and delays in cash transfers eroding participants’ confidence; conditional cash transfers requiring all household members to participate being poorly achieved; and high risk patients (e.g. the formerly incarcerated, the homeless, and those with drug or alcohol addiction) being difficult to engage and thus benefiting least from the intervention.

#### Conditional vs unconditional

##### Challenges

The effectiveness and cost-effectiveness of conditional versus unconditional cash transfer schemes is under debate. For example, conditionality does not seem to be essential to meet HIV-related objectives [26, 47]. Furthermore, the implementation of conditionality to achieve public health objectives, including those related to TB programmes, may be hampered by existing administrative and health system weaknesses. Therefore, cash transfer programme interventions may not have the financial or administrative capacities to monitor the health-seeking behaviour conditionality or offer adequate health systems infrastructures to make these conditions feasible or ethical [6, 48–50]. For example, in settings with limited TB care services, implementing conditional cash transfer schemes to improve treatment TB adherence may have limited impact if treatment or access to treatment is not consistently available.

Conditionality may also raise important questions concerning choice, autonomy and decisional capacity of the poor without due flexibility determined by cultural and socioeconomic factors [48]. Conditionality may stimulate poor people to seek TB care while neglecting other important health priorities in the household such as maternal/child health or to act against their common belief or will [51, 52]. Evidence concerning this is inconsistent: the low uptake of the Janani Suraksha Yojani programme in India among the poorest and least educated women demonstrates that conditioning the cash transfers do not necessarily overcome cultural or geographical barriers that hamper people’s capacity to meet behavioural conditions [14]. Conversely, in Malawi, monetary incentives of less than a tenth of a day’s wage dramatically changed people’s health seeking behaviours by compensating the psychological and economic costs of HIV testing and reduced the gender gap in accessing HIV services [53]. Thus, cash incentives or enablers for seeking TB care may compensate for the direct material and the indirect psychological costs of TB diagnosis and ultimately increase gender equity in access to TB diagnosis [53]. In conclusion: a) There is currently no direct, rigorous evidence to guide to guide the circumstances under which TB-related cash transfer interventions should be conditional and if so, on what TB-related behaviours conditionalities should be applied and how stringently. b) Even evidence from the HIV/AIDS experience is not conclusive and the issue has not yet been rigorously explored [13]. Finally, c) the effectiveness of utilizing conditionality in cash transfers depends on the outcome under evaluation, the feasibility of the condition, the program context, and the target population [34, 54, 55].

##### Solutions

Given the numerous knowledge gaps, the strict application of conditionality to increase TB case detection and treatment success rate may be unjustified in countries with limited administrative and health care capacities. In these settings, an option may be a “soft” form of conditionality in which conditions are simple or made less stringent (e.g. penalties for non-adherence do not imply the exclusion of participants from the programme) [48, 55]. Another possibility may be to indirectly encourage the attendance of TB care services by making the cash transfer conditional upon the attendance of workshops and training sessions concerning TB that may help TB-affected families to overcome the fear and the stigma of approaching the TB services.

Should conditionality be deemed appropriate, it may be useful to assess the features of the local TB epidemic and available care. This may be accomplished with qualitative research with TB-affected families and main TB stake-holders to test the feasibility and acceptability of conditionalities from the target population and to design them so to meet better the specific challenges experienced by TB-affected families.

#### Targeting approach

##### Challenges

Cash transfer programs may be made TB-sensitive by targeting people at high risk of TB, or may be transformed to TB-inclusive programs by specifically including TB patients among their target population. These programs may broadly target TB-patients and/or their households; restrict their target to TB-patients subgroups (e.g. drug-resistant TB, TB-HIV co-infected or TB patients affected by other co-morbidities such as mental health issues or diabetes); or restrict to TB-patients during their highest risk time period (e.g. the time period when treatment loss to follow up or onward TB transmission is most likely).

Depending on the targeting strategy, issues concerning accuracy, costs, cost-effectiveness, equity, fairness, sustainability, unintended consequences and stigma may arise: for instance, using TB as a targeting criterion may be more efficient, but could be stigmatising. Furthermore, it may foster perverse incentives in which patients attempt to remain sick assuming that this may entitle them to receive the cash benefits. This concern was specifically raised by TB care providers in the intervention from Lutge et al. [43], although this was not observed and conversely patients receiving vouchers more often tended to be more likely to achieve treatment success [44].

Conversely, “passive” targeting, based only on poverty criteria, may be inefficient or impractical. For example, a 2010 analysis in Brazil cross-linking the national TB registry with the Single Registry for Social Programmes (a census database to identify people enrolled in social protection programmes in Brazil) showed that only one fourth of TB patients who live below the poverty line are already enrolled in government’s social protection programmes, with only 14 % of them being amongst the beneficiaries of Bolsa Familia. This unexpectedly low overlap between the two data sources suggest that in Brazil targeting based only on socioeconomic criteria may be insufficient to capture most TB patients in needs of social protection for a number of reasons discussed by Torrens and colleagues [29]. Moreover, even if such poor families are enrolled in Bolsa Familia, it is often the case that they do not have the necessary requirements to actually receive the transfers (e.g. a billing address, identification documents, or proof of income documents) [29, 56]. In contrast, in Zambia targeting for vulnerability to HIV based on poverty levels may be considered a viable option to also capture a substantial proportion of households affected by TB as well as HIV that are in need of financial support that avoids stigmatisation [38].

##### Solutions

There is very limited evidence to inform the best targeting strategy for cash transfer interventions aiming at enhancing TB elimination; however the most suitable approach seems to be highly setting-specific and pretty much dependant on the TB issues to be addressed. For example, in high-TB burden countries preventing TB transmission may require targeting people at high-risk for TB; on the other hand, in settings where TB priorities are more circumscribed (i.e. addressing TB-related catastrophic costs, enhancing TB treatment adherence), and/or the epidemic is restricted to selected groups of the population (i.e. drug users, homeless, prisoners) then it may be more beneficial to target directly TB-affected households or TB patients.

As a general rule, cash transfer interventions, and more broadly social protection initiatives, should be delivered in way to be non-stigmatising, non-discriminatory and aim to optimise inclusiveness and equity [35]. In order to achieve this ultimate goal, counties are often encouraged to use multiple criteria [48] such as multidimensional targeting that identifies beneficiaries based on the objectives of the intervention and various forms of deprivation affecting the target population [57]. In the case of TB-sensitive or TB-inclusive cash transfer intervention, a multiple targeting strategy could include a combination of the following criteria: A. living in extreme poverty as assessed by econometric or asset-based indices; B. not having household members able to work; C. not having other forms of social assistance; and D. the household TB history or a high household risk score based on TB risk factors (such as household prevalence of HIV, alcohol abuse, smoking, diabetes) [48].

#### Sustainability

##### Challenges

Data concerning existing cash transfers costs are limited, not always comprehensive, and lacking for low compared to middle income countries. Where available, evidence suggests an average cost between 12.5 US$ and 26.5 US$ per month per beneficiary (with 4 to 20 % of the monthly household consumption expenditure of the beneficiaries covered), which represents from 0.36 to 1.54 % of GDP for any given implementing country [24, 58]. Analyses of costs of social protection packages generally conclude that social protection is affordable in most countries, including deprived settings such as Sub Saharan low income countries, as long as costs are less than 1 % of countries’ GDP share [58, 59]. In Zambia, for example, the annual costs of the three pilot studies for the national unconditional cash transfer were US$40-$82 million [38]. A 2009 analysis showed that even if a pilot in the Katete district was expanded throughout the country to target 450,000 beneficiaries with transfers of an average of US $15/month, this would represent only 2.4 % of the Zambian National budget or 0.7 % of the GDP in 2007 [38]. These data suggest that implementing the Social Cash Transfer scheme on a national scale in Zambia is likely to be affordable. However public spending in Zambia on social assistance represents <0.1 % of GDP [38] suggesting that scaling up the Social Transfer Scheme across the country requires increased financial investment, but also political will [37].

It remains also unclear whether economic assessments should distinguish between different types of costs (e.g. administrative versus transfer costs) as well as distinguish between programs supported by aid provided by international donors versus domestic funding. Generally, government-led schemes are considered to have greater long-term sustainability [37, 38]. However, it is not yet clear how many government-led cash transfer programs may have the financial and logistic resources to sustainably encompass specific public health objectives, such as TB [60]. The incorporation of TB beneficiaries in existing cash transfer initiatives or linking them with TB activities is likely in fact to require additional costs for setting up new operating procedures, staff training and the creation of new collaborative forms of social and medical management. Countries may not be able to afford these costs without mobilizing extra-funds. In these circumstances, funding sources may be hesitant to sponsor collaborative projects if these payments may interfere with the performance and objectives of the original programme [60].

##### Solutions

Cost concerns are legitimate; Referring to TB specifically, globally there are about 9 million new cases of TB each year. Hypothetically, even if $100 worth of social protection were to be additionally provided to every patient globally then the resultant expense of would be approximately $900 million. This is not a modest expense in the current global TB investments. However, costs should be evaluated through a proper extended cost-effectiveness analysis (ECEA), which accounts not just for the health gain against the cost of an interventions, but also the financial protection achieved as a result of that intervention (both in terms of poverty aversion and equity improvement) [61, 62]. In the case of TB, the potential financial benefit arising from proper control measures seems considerable: ecological studies have reported an effect of 0.2–0.4 % decreased economic growth for every 10 % higher TB incidence [63]. This corresponds to an annual loss of US$1.4-2.8 billion in economic growth worldwide. In addition, the World Bank estimates that loss of productivity attributable to TB is 4-7 % of some countries' GDP [61]. In a more recent study, Verguet and colleagues have positioned TB treatment among the best interventions at preventing medical impoverishment (i.e. 96 cases of poverty averted per every US$100,000 spent) [62].

At the micro-level the impact of cash transfers is expected to be even more significant: in most settings the average costs encountered by TB sufferers exceed 10 % annual household expenditures [64]. In worst cases this burden can exceed 20 %, a threshold that has been defined as “catastrophic” not only because associated with a further impoverishment of TB-affected households, but also associated with an increased risk of TB mortality, treatment loss to follow up, or TB recurrence [65]. Cash transfers can reduce financial vulnerability and potentially improve TB treatment adherence and outcome [65]. Furthermore, it should be considered that even TB-specific cash transfer interventions are likely to achieve multiple health and development impacts, not just TB [12, 65]. The capacity to produce multiple benefits is the strongest argument to propose co-financing mechanisms through which various sectors pool funds or engage in joint budgeting to fund interventions with multi-sectoral benefits [12, 66]. Under this approach several models have been proposed: one may be to consider the TB-related costs separately from the core services of the original intervention. International and/or domestic donors may be willing to specifically cover these marginal TB-related costs [18, 66, 67]. Alternatively it may be worth exploring the creation of a partnership among donors as theorised in the UNAIDS Investment framework to realise health and development funding synergies [68].

### A research road map for the future

The research priority actions to support and inform the new End TB strategy are listed in Table 2 and summarised here.

A significant scientific and financial investment is urgently needed to fill knowledge gaps, the most important of which is whether cash transfers can enhance the accomplishment of the goals and targets of the new End TB strategy. Impact and operational evidence can be generated both by: i) replicating TB-specific initiatives such as CRESIPT in different settings with the objective of creating a network of projects sharing methodological and programmatic lessons, and ii) undertaking quasi-experimental as well as natural experiments to understand what is the unintentional impact of cash transfer interventions on TB epidemiology. This latter approach is heavily dependent on the possibility of linking TB and cash transfer interventions datasets. Alternatively, appropriate proxies or biomarkers for TB and other respiratory infections could be identified in future to replace the actual measurement of TB indicators to determine whether a tangible impact on these indicators can be observed and - if so - how much this could be attributed to the intervention.

The first and second approach can collectively contribute to inform the design of more effective and cost-effective TB-sensitive cash transfer interventions. The pathway towards TB-sensitive interventions may also benefit from: i) the development of a programmatic assessment tool for countries to map the existing social protection platforms and to inform the decision on which implementation strategy, TB-specific, TB-inclusive or TB-sensitive, may be the most suitable in their own context given the local social protection and TB epidemic conditions; ii) the inventory or mapping of existing cash transfer initiatives and assessment of the extent for potential overlap with TB control activities with the ultimate aim to identify the most promising initiatives that could be most effectively and cost-effectively adapted to meet TB control objectives; iii) the development of metrics to measure economic impact of TB for households [69, 70]; and iv) the development of rapid and relatively inexpensive assessment methods such as mathematical modelling techniques to predict the effectiveness and cost-effectiveness of a given intervention under different operational conditions and after controlling for local TB and socioeconomic trends.

To inform the new End TB strategy, research studies need to go beyond the evaluation of costs and provide a more in depth understanding of critical operational aspects. *Ad hoc* studies should be undertaken to assess the importance of conditionality and answer questions such as: what are the determinants of success of conditionality? Is the compliance to conditionality influenced by the cash transfer size, frequency of delivery and other psychosocial determinants? Does the TB status (i.e. sensitive or drug-resistant TB, loss to follow up or relapse case) as well as the stage of disease (TB infection, disease) affect the success of conditionality? How do we effectively and cost-effectively monitor conditionality?

Efforts should be made to understand the relative advantage of different targeting strategies and to assess the accuracy of targeting. Finally a thorough assessment of costs and the identification of innovative funding mechanisms seem important to justify sustainable investments in cash transfer interventions for ending TB.

## Conclusions

In this paper we have reviewed four major operational aspects to consider if implementing cash transfer interventions as part of a country TB response, including programme design, conditionality, targeting and sustainability. Most of these challenges apply to all cash transfer interventions, but we appraised them through the lens of TB epidemiology and control. By doing so we aimed to foster discussion and stimulate research in this field within the TB scientific community. We took a broad approach but have not covered certain important aspects such as the ethical dimension of using cash incentives for health promotion purposes; however this paper has the potential to represent a useful entry point for TB control implementers and researchers to introduce them to new concepts and definitions and the discourse on cash transfer interventions.

Our paper demonstrates that there are important lessons that can be inferred from the literature and usefully – albeit provisionally – applied to TB. However crucial knowledge gaps remain that can be only partially inferred from the increasing experience gained from cash transfer initiatives implemented for other diseases such as HIV/AIDS.

While the formulation of formal policy recommendations is premature at this stage, this does not justify inaction or complacency. A clear research agenda is needed to inform and support the End TB strategy; however, equally important will be the political commitment and the financial support of all the key stakeholders towards this goal.

## Abbreviations

CRESIPT, community randomised evaluation of a socioeconomic intervention for the prevention of tuberculosis; ECEA, extended cost-effectiveness analysis; GDP, gross domestic product; TB, Tuberculosis; UNDP, United Nations Development Programme; WHO, World Health Organization
